# Rapidly Progressing Melioidosis Outbreak in City Center Zoo, Hong Kong, 2024

**DOI:** 10.3201/eid3109.250823

**Published:** 2025-09

**Authors:** Christopher J. Brackman, Ivan Tak-Fai Wong, Allen S.L. Chan, Patrick C.K. Pun, Dorothy Hong-Ting Cheung, Anne C.N. Tse, Carlton P.M. Yuen, Pierra Y.T. Law, Wing-Yin Tam, Franklin Wang-Ngai Chow, Gilman Kit-Hang Siu, Thomas Hon-Chung Sit

**Affiliations:** Government of the Hong Kong Special Administrative Region, Hong Kong, China (C.J. Brackman, A.S.L. Chan, P.C.K. Pun, A.C.N. Tse, C.P.M Yuen, P.Y.T. Law, T.H.-C. Sit); Polytechnic University, Hong Kong (I.T.-F. Wong, D.H.-T. Cheung, W.-Y. Tam, F.W.-N. Chow, G.K.-H. Siu)

**Keywords:** melioidosis, Burkholderia pseudomallei, bacteria, zoonoses, zoo animals, primates, phylogenetic analysis, One Health, outbreak, Hong Kong, China

## Abstract

In October 2024, twelve primates from 4 species died of sepsis at the Hong Kong Zoological and Botanical Gardens. Postmortem examinations and microbiological analyses confirmed *Burkholderia pseudomallei* infection. Phylogenetic analysis revealed a clonal sequence type 46 strain with minimal variation, signifying a single source. This outbreak highlights melioidosis risk in zoo settings.

During October 2024, a series of sudden primate deaths occurred in the mammals section of the Hong Kong Zoological and Botanical Gardens (HKZBG), a popular city-center tourist attraction in Hong Kong, China, managed by the Leisure and Cultural Services Department. At the time of the outbreak, the facility housed 80 nonhuman primates (NHPs) across 12 species. We report the clinical course of illness and outcomes for the outbreak.

## The Cases

On the morning of October 13, 2024, four monkeys at HKZBG were found dead in their enclosures: 1 cotton-top tamarin (*Saguinus oedipus*), 2 white-faced sakis (*Pithecia pithecia*), and 1 common squirrel monkey (*Saimiri sciureus*). Concurrently, another 2 cotton-top tamarins, 1 white-faced saki, and 1 De Brazza’s monkey (*Cercopithecus neglectus*) with clinical signs of varying severity (e.g., lethargy, anorexia, fever) were sent to the zoo clinic for examination and emergency care; all 4 monkeys died later that day. Upon observed clinical signs, 1 additional white-faced saki and 1 De Brazza’s monkey were isolated for intensive monitoring; the saki died on October 14 and the De Brazza’s monkey on October 22. Another 2 common squirrel monkeys hospitalized on October 16 died on October 19 and October 20 (Table; Appendix Figure 1).

**Table:** Characteristics and laboratory findings for a rapidly progressing melioidosis outbreak in city center zoo, Hong Kong, 2024*

Case no.	Species	Age, y/sex	Onset of clinical signs	Date of death	*B. pseudomallei* isolated (source)
Case 01	White-faced saki	2/M	Found dead	2024 Oct 13	Yes (lung)
Case 02	Common squirrel monkey	18/M	Found dead	2024 Oct 13	Yes (liver, lung, spleen)
Case 03	White-faced saki	24/F	2024 Oct 12	2024 Oct 13	Yes (liver, kidney, lung, spleen)
Case 04	White-faced saki	31/F	2024 Oct 13	2024 Oct 13	Yes (liver, lung, spleen)
Case 05	Cotton-top tamarin	3/M	Found dead	2024 Oct 13	No
Case 06	Cotton-top tamarin	13/M	2024 Oct 13	2024 Oct 13	Yes (lung)
Case 07	De Brazza’s monkey	11/M	2024 Oct 12	2024 Oct 13	Yes (liver, lung, spleen)
Case 08	Cotton-top tamarin	1/M	2024 Oct 13	2024 Oct 13	Yes (liver, kidney, lung, spleen)
Case 09	White-faced saki	1/F	2024 Oct 13	2024 Oct 14	Yes (lung)
Case 10	Common squirrel monkey	6/F	2024 Oct 15	2024 Oct 19	Yes (liver, lung, spleen, brain)
Case 11	Common squirrel monkey	10/F	2024 Oct 15	2024 Oct 20	Yes (spleen)
Case 12	De Brazza’s monkey	13/F	2024 Oct 13	2024 Oct 22	Yes (liver, spleen)

*All animals had *Burkholderia pseudomallei* detected by real-time PCR in liver or spleen tissue.

In total, 12 monkeys spanning 4 species eventually died in this outbreak. Species-specific mortality rates were high: 2 (50%) of 4 De Brazza’s monkeys, 3 (75%) of 4 common squirrel monkeys, 3 (50%) of 6 cotton-top tamarins, and 4 (36.4%) of 11 white-faced saki died. Collectively, the deaths represented 15% of the zoo’s NHP population and a 48% mortality rate among the 25 animals comprising the 4 species, underscoring the outbreak’s severity.

To safeguard public and animal health, the mammals section of the zoo was temporarily closed starting on October 14. All enclosures were thoroughly cleaned and disinfected. The remaining animals in the section were clinically healthy. Health monitoring for staff who take care of animals was provided, and health conditions of staff were unremarkable.

**Figure 1:**
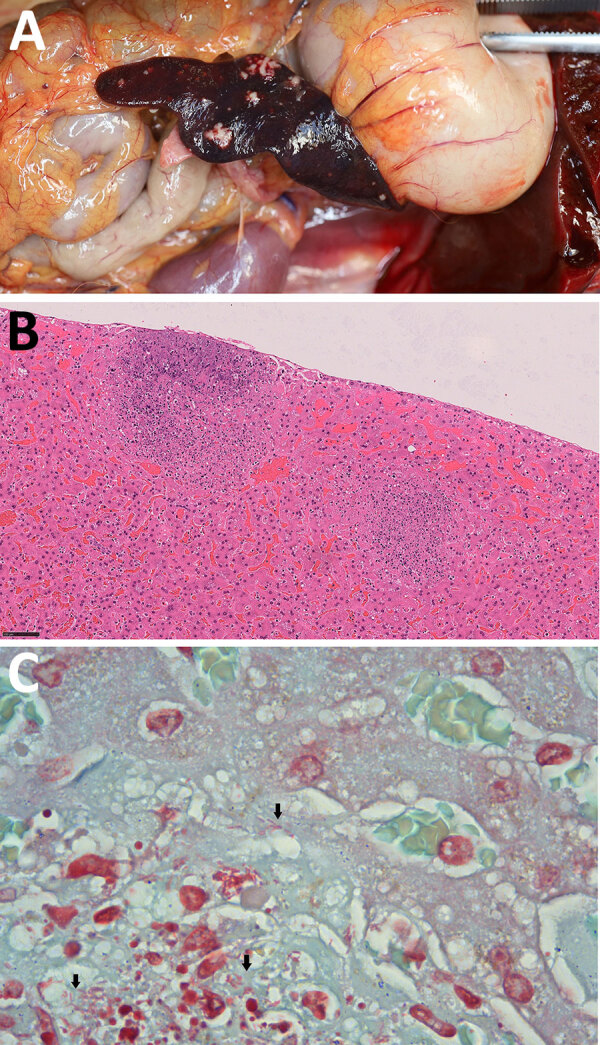
Gross and histopathological features of nonhuman primates who died during rapidly progressing melioidosis outbreak in city center zoo, Hong Kong, 2024. A) Gross pathology of splenic lesion in a white-faced saki (*Pithecia pithecia*). The spleen exhibits multifocal to coalescing necrosis. B) Necrotizing hepatitis of the liver in a cotton-top tamarin (*Saguinus oedipus*). Hematoxylin and eosin stain; original magnification ×200. C) Numerous gram-negative rods (black arrow) at the areas of necrosis in the liver of a cotton-top tamarin. Gram stain; original magnification ×1,000 with oil immersion objective.

A government working group conducted comprehensive follow-up actions, including postmortem examinations and diagnostic testing, to investigate the monkey deaths. On October 18, laboratory results confirmed that all tested monkeys had died from sepsis caused by *Burkholderia pseudomallei* infection. The HKZBG veterinarian performed postmortem examination and tissue sampling of the 8 monkeys that initially died on October 13. The remaining 4 monkeys were sent to the Tai Lung Veterinary Laboratory under the Agriculture, Fisheries and Conservation Department of the Government of the Hong Kong Special Administrative Region for postmortem examination, histopathology, and microbiological testing. Gross and histopathologic findings of all animals demonstrated that the liver and spleen were the most severely affected organs, characterized by acute necrotizing to necrosuppurative splenitis (Figure 1, panel A) and hepatitis (Figure 1, panel B); intralesional gram-negative bacilli were detected (Figure 1, panel C). Evidence of hematogenous spread to the lungs was also present in some monkeys, resulting in mild fibrinonecrotic interstitial pneumonia.

Bacterial cultures yielded 11 pure isolates, which were initially identified as *Burkholderia* spp. using a Biotyper matrix-assisted laser desorption/ionization time-of-flight mass spectrometry system (Bruker, https://www.bruker.com). Real-time PCR targeting a 115-bp fragment of the type III secretion system confirmed *B. pseudomallei* (*1*). Additional molecular screenings for monkeypox virus, coronavirus, SARS-CoV-2, *Leptospira* spp., and influenza A virus all produced negative results.

Initial investigations hypothesized an environmental source, including possible release from recent soil disturbances in early October. A comprehensive environmental assessment was conducted, including PCR and culture of 25 soil, 27 drinking water, 10 feed supplement, and 8 environmental samples. All samples tested negative for *B. pseudomallei* (Appendix).

*B. pseudomallei* was cultured from the liver, lungs, or spleen of 11 of the 12 dead animals. The isolates underwent whole-genome sequencing (Appendix), and a hybrid assembly approach using Hybracter version 0.11.0 (https://github.com/gbouras13/hybracter) generated high-quality, closed-gap complete genomes (*2*). Multilocus sequence typing (MLST) based on profiles from the PubMLST database (https://pubmlst.org) classified all isolates as sequence type (ST) 46 and core-genome MLST (cgMLST) type 1070 (*3*).

For phylogenetic context, we constructed a maximum-likelihood phylogeny comparing study isolates to 40 reference *B. pseudomallei* genomes representing ST46 and closely related sequence types from GenBank (*4*). The tree used single-nucleotide polymorphisms (SNPs) derived from 3,127 single-copy cgMLST genes (*5*) (Appendix). Results showed that the 11 Hong Kong isolates clustered together in a monophyletic clade with 100% bootstrap confidence (Appendix Figure 2), exhibiting exceptionally tight genetic relatedness of only 0–1 core-genome SNP (cgSNP) differences. In addition to core-genome phylogeny, whole-genome average nucleotide identity analysis further confirmed high genetic similarity (99.99839%–99.99954%) among the 11 isolates (Appendix Figure 3). Considering that *B. pseudomallei* can develop 8 SNPs during a 12-day acute infection period (*6*), the minimal SNP variation among the HKZBG isolates suggests a single clonal infection source (*7*), likely from a singular introduction event rather than sustained transmission among the animals.

**Figure 2:**
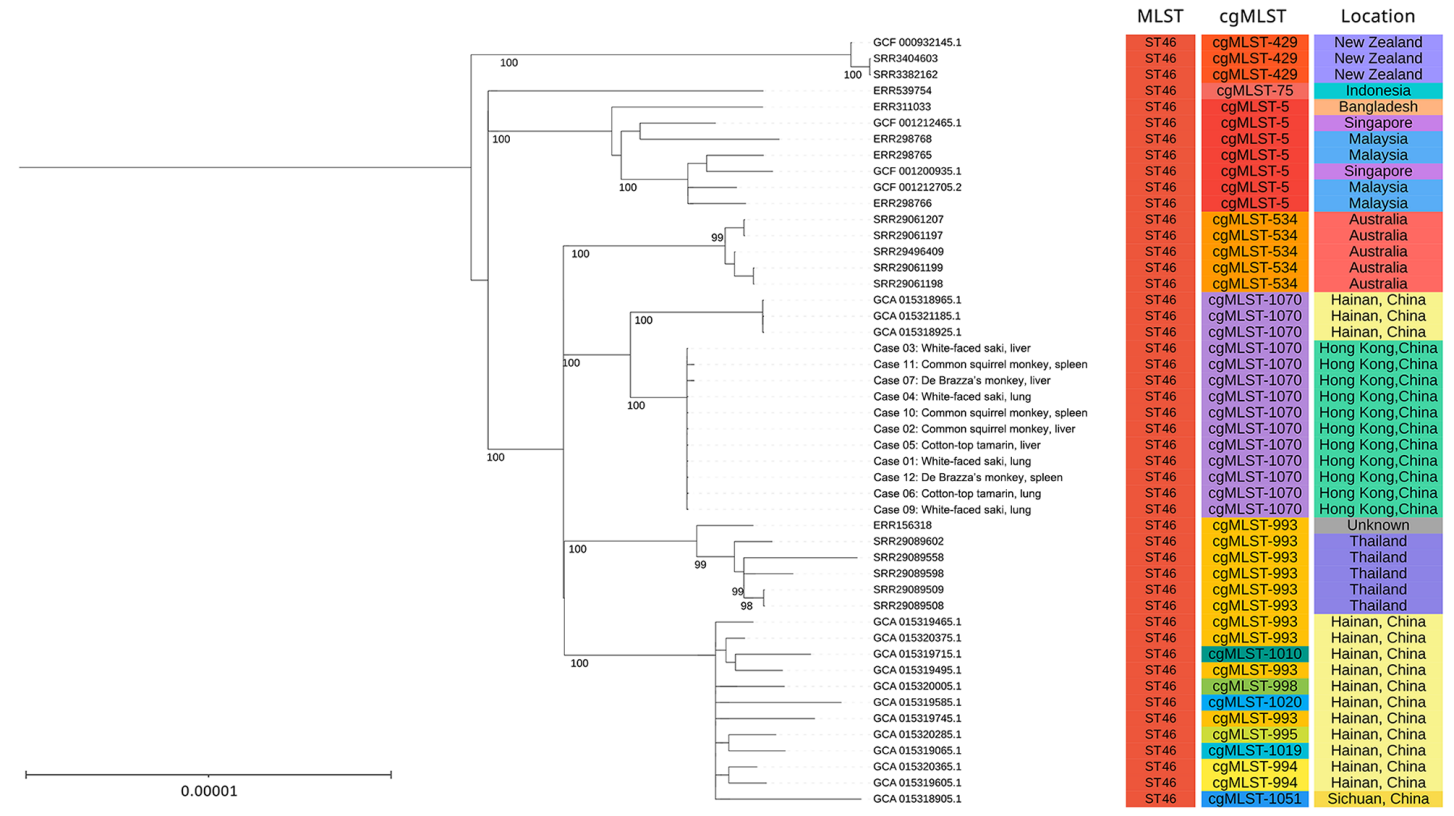
Phylogenetic analysis of *Burkholderia pseudomallei* ST46 genomes from nonhuman primates that died during rapidly progressing melioidosis outbreak in city center zoo, Hong Kong, 2024. Tree compares case nos. 01−11 with 40 reference genomes acquired globally from NCBI. Maximum-likelihood phylogenies of the single-nucleotide polymorphisms of the 3,127 single-copy cgMLSTs are shown. Bootstrap analysis of 1,000 replicates was performed, and bootstrap values of selected nodes are shown. The tree is midpoint rooted. The 11 monkey isolates formed a monophyletic clade with 100% bootstrap confidence. Distinct colors are used as a visual aid to group isolates by their MLST, cgMLST, and geographic location. Scale bar indicates nucleotide substitutions per site. cgMLST, core-genome MLST; MLST, multilocus sequence typing; ST, sequence type.

**Figure 3:**
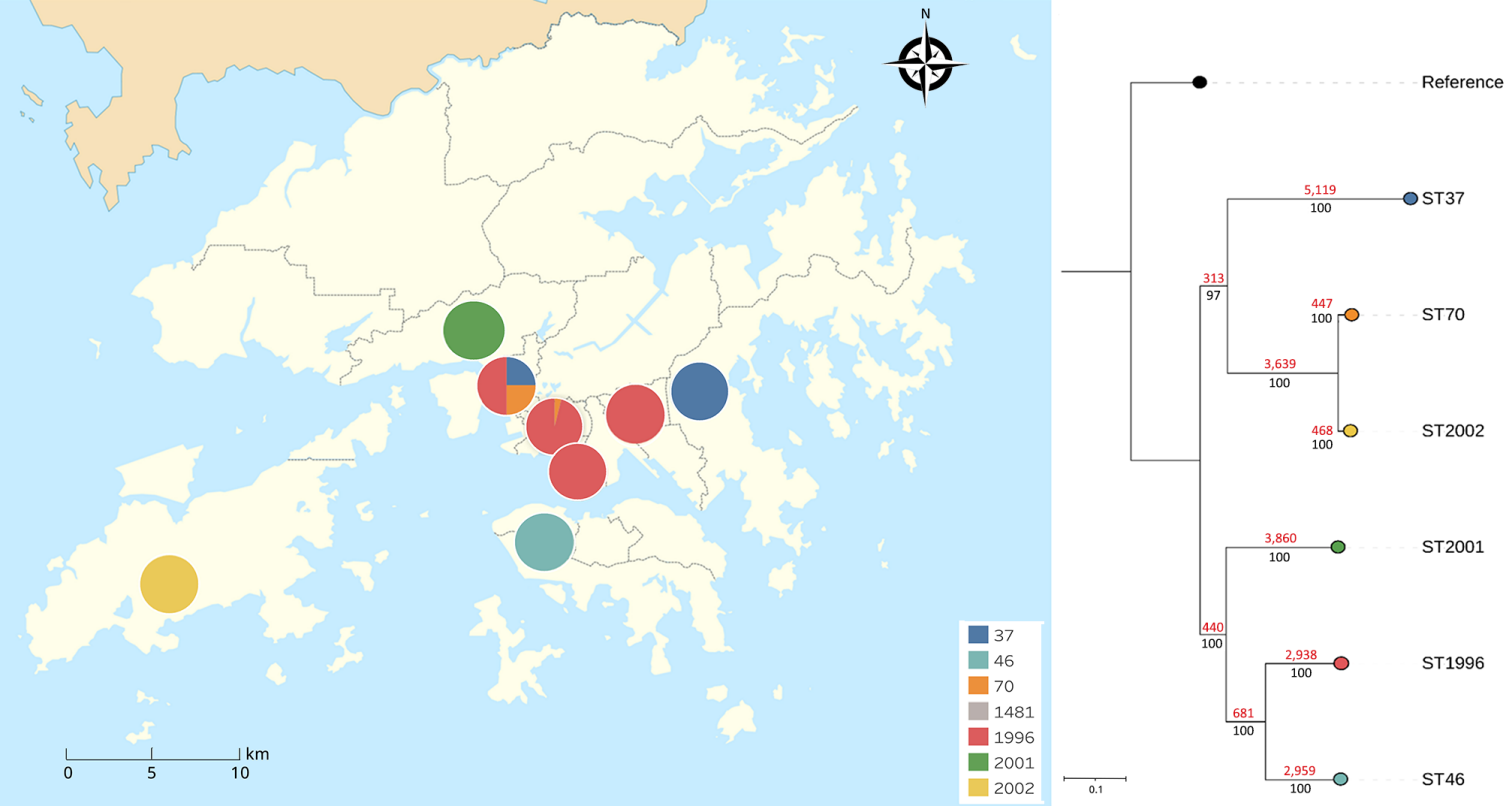
Spatial distribution and genetic relationships among *Burkholderia pseudomallei* STs reported in China and a rapidly progressing melioidosis outbreak in city center zoo, Hong Kong, 2024. Colored circles on map show locations where different STs were reported; sections indicate relative percentages of each ST in each location. Red numbers on the phylogenetic tree indicate core-genome single-nucleotide polymorphism differences between strains; black numbers represent bootstrap values. The tree is midpoint rooted. The reference genome used for comparison is a pseudogenome constructed by concatenating the first allele of each core-genome multilocus sequence typing gene available in the PubMLST database (https://pubmlst.org). Scale bar indicates nucleotide substitutions per site. ST, sequence type.

Focused comparative analysis of ST46 (Figure 2) revealed that the HKZBG clade was most closely related to a clonal cluster of 3 strains from northern Hainan Province, China, with a genetic distance of 18 cgSNPs. The next closest relatives were strains from Australia (27 cgSNP difference) and Thailand (31 cgSNP difference). Such minimal divergence underscores that ST46 is a recurring sequence type within the Asia–Oceania region. Although that ST is the third most frequently reported in the global PubMLST database and has been isolated from humans, the environment, and other animals, including monkeys (*8*,*9*), it had not been previously reported in Hong Kong. Moreover, the isolates from this outbreak are genetically distinct from the local outbreak strain ST1996 reported in 2022 (*10*) and from other local sequence types, such as ST70, ST37, and ST32 (*11*) (Figure 3). Among globally reported ST46 strains, the closest relatives to the HKZBG clade were strains isolated in 2002–2003 from northern Hainan Province, China (National Center for Biotechnology Information Assembly database accession nos. GCA_015312861.1, GCA_015312871.1, and GCA_015312851.1) (Figure 2). Those isolates share the same cgMLST type 1070 profile, suggesting that this lineage has been established in southern China for decades (*12*).

## Conclusions

The rapid and nearly simultaneous deaths of multiple primates, together with the swift progression of the disease, suggest that this melioidosis outbreak was the result of a concentrated or highly virulent exposure event (*13*). Postmortem findings revealed extensive hepatic and splenic involvement in all animals. Although some animals exhibited pneumonia, pulmonary lesions were mild, and the pattern was characteristic of hematogenous spread to the lungs rather than bronchogenic. That finding is different from the lesions observed in NHP models after aerogenous infection (*14*,*15*). 

Despite extensive investigations, including environmental sampling and genomic analysis, the precise source of infection in this outbreak remains unidentified. The initial hypothesis that soil disturbances released environmental *B. pseudomallei* was not supported because of negative environmental results and because most affected monkeys had long-term residency at the zoo (many >6 years) with no history of melioidosis.

In reaction to this incident, the zoo implemented stringent biosecurity measures, including thorough enclosure disinfection and restricted access to affected areas. No further monkey deaths were recorded after October 22, and no cases of human melioidosis were noted during the investigation period.

This outbreak, which resulted in the loss of 12 monkeys, including critically endangered cotton-top tamarins, highlights the potential threat of melioidosis in zoologic settings. Climate change potentially could increase the incidence of *B. pseudomallei* infections, even in urban environments like Hong Kong, requiring enhanced biosecurity, vigilant health monitoring, and a high index of suspicion for melioidosis in cases of unusual illnesses and deaths in captive wildlife. Such proactive measures are critical for protecting both animal and human health.

Appendix Additional information for rapidly progressing melioidosis outbreak in city center zoo, Hong Kong, 2024.
