# Supplementary material for: Rapidly Progressing Melioidosis Outbreak in City Center Zoo, Hong Kong, 2024
Source: Emerg Infect Dis. 2025 Sep;31(9):1815–9. doi: 10.3201/eid3109.250823 (PMC12407217; doi:10.3201/eid3109.250823)
Supplement: Appendix — Additional information for rapidly progressing melioidosis outbreak in city center zoo, Hong Kong, 2024. [file 25-0823-Techapp-s1.pdf]

Article DOI: https://doi.org/10.3201/eid3109.250823

EID cannot ensure accessibility for supplementary materials supplied by authors. Readers who have difficulty accessing supplementary content should contact the authors for assistance.

Rapidly Progressing Melioidosis Outbreak in City Center Zoo, Hong Kong, 2024

Appendix

Supplemental Methods

Environmental Sampling and Testing

A comprehensive environmental investigation was conducted to identify the potential source of the outbreak. A total of 70 environmental and food/water samples were collected. This included 25 soil samples collected from the soil digging site (n = 3), surrounding flower beds (n = 17), and planters inside affected enclosures (n = 5). Additionally, 8 environmental swabs were taken from feed and water troughs, and 10 feed/supplement samples and 27 water samples were collected for analysis.

All 70 samples were sent to the Public Health Laboratory Centre (PHLC) and subjected to molecular testing via a *Burkholderia pseudomallei*-specific PCR assay. In parallel, the 25 soil samples were also processed for bacterial culture by plating on Ashdown's agar.

Bacterial Isolates

Burkholderia pseudomallei (BPS) was isolated from necropsy samples preserved in Microbank beads and transported to the Hong Kong Special Administrative Region, People’s Republic of China Polytechnic University (PolyU) at 4°C using triple packaging. Upon arrival, samples were subcultured in a Class II biosafety cabinet onto Ashdown's agar, which selectively promotes *Burkholderia* spp. growth. Colonies exhibiting characteristic wrinkled morphology and metallic sheen were initially presumed to be BPS.

Initial Isolate Identification by MALDI-TOF

Species identification was performed using matrix-assisted laser desorption/ionization time-of-flight (MALDI-TOF) mass spectrometry (MALDI Biotyper, Bruker Daltonics; IVD

version 4.2.90.7). The initial identification as *Burkholderia* spp. reflects a known limitation of the standard Bruker Biotyper database, which does not include *B. pseudomallei* (*1*). This commonly results in a genus-level identification or a misidentification as a closely related species. For this reason, all presumptive isolates were forwarded for definitive molecular confirmation.

PCR Protocol for Clinical Isolate Identification

Confirmation of *B. pseudomallei* was achieved through Real-time PCR targeting the Type III secretion system, specific to *B. pseudomallei*, ensuring accurate identification of the pathogen (*2*). The assay was designed to amplify a 115-bp fragment of the *Burkholderia pseudomallei* type III secretion system gene cluster (GenBank accession no. AF074878). The primer and probe sequences used were as follows:

- **Forward Primer (BpTT4176F):** 5′-CGTCTCTATACTGTCGAGCAATCG-3′
- **Reverse Primer (BpTT4290R):** 5′-CGTGCACACCGGTCAGTATC-3′
- **Probe (BpTT4208P):** 5′-CCGGAATCTGGATCACCACCACTTTCC-3′

The TaqMan probe was labeled with a 6-carboxyfluorescein (FAM) reporter dye at the 5′ end and a Black Hole Quencher 1 (BHQ1) at the 3′ end.

The PCR was performed in a 15 µL reaction volume containing 5x LightCycler Multiplex DNA Master, 10 µM each of the forward and reverse primers, and a 4 µM TaqMan probe. The thermal cycling conditions were as follows: an initial denaturation at 95°C for 2 minutes, followed by 45 cycles of 95°C for 5 seconds and 59°C for 40 seconds, and a final cooling step at 40°C for 30 seconds.

Nucleic Acid Extraction and Sequencing

Total nucleic acid was extracted from isolates using the QIAamp BiOstic Bacteremia DNA Kit. Isolates were sequenced on an Illumina NovoSeq 6000 platform (Illumina Technologies, San Diego, CA, United States) at HaploX. This generated 150bp paired-end reads totaling 1Gb per isolate. For long-read sequencing, libraries were constructed with the Rapid Barcoding Kit 96 (SQK-RBK110.96) using 800ng of normalized library per isolate. Sequencing was performed on GridION MK1 with R10.4.1 flow cells (ONT, UK) using the Dorado

basecaller v7.3.9 with super-accuracy basecalling (SUP). This generated 1Gb of sequence per isolate as FASTQ files.

Hybrid Assembly

Hybracter (v0.11.0) hybrid assembly approach was used to generate high-quality genome assemblies (*3*). In brief, reads were quality controlled with Filtlong, Porechop and fastp. Long reads were assembled into contigs using Flye, polished with Medaka for two rounds, and finally polished with short reads using Polypolish and Pypolca.

MLST and cgMLST Analysis

Multilocus sequence typing (MLST) was performed to assign a sequence type (ST) to each isolate. The sequences of the seven MLST alleles (*ace, gltB, gmhD, lepA, lipA, nark, ndh*) were queried for a match to the existing sequence types on the MLST database (*4*). The ST was assigned based on the MLST allele profile available on PubMLST (*5*). Core-genome MLST (cgMLST) analysis was also conducted by extracting the 4090 cgMLST alleles from PubMLST to characterize isolates at a high-resolution (*5*).

Average Nucleotide Identity Analysis

Average Nucleotide Identity (ANI) was employed to assess the genetic similarity among the BPS ST46 genomes using ANIClustermap (*6*). This analysis encompassed 11 HKZBG isolates compared against 38 ST46 genomes sourced from NCBI datasets. These reference genomes, which were detailed in a previous study (*7*), originated from diverse geographic regions including Oceania, Southeast Asia, and People's Republic of China. For the analysis, ANI calculations for all-vs-all genome comparisons were conducted using fastANI, and the results were visually represented in a cluster map generated using the seaborn library.

Core Gene SNP Phylogenetic Analysis

The 4090 cgMLST genes of *B. pseudomallei* were downloaded from PubMLST and concatenated into a single nucleotide sequence alignment to serve as a reference genome (*5*). For the phylogenetic analysis, 70 *B. pseudomallei* genomes were selected from the NCBI assembly database. These genomes were chosen based on their similarity in STs to the outbreak isolates and the presence of exactly two chromosomes. Additionally, these genomes were identified in previous studies as genetically close and classified within the same group as ST46 (*7*). The selected genomes represent strains from diverse geographic regions including Oceania, Southeast

Asia, Russia, and People's Republic of China. All study and reference genomes were aligned to the cgMLST pseudogenome using Snippy v4.6.0 with default settings (*8*). This alignment was performed to identify 14,015 SNP profiles across the 3,127 single-copy cgMLST genes shared among all study isolates. The maximum likelihood phylogeny of the core alignment was estimated using IQ-TREE v2.3.0 (*9*). A GTR+F+I model and 1000 regular bootstrap replicates were adopted for this analysis. The final tree was mid-point rooted and annotated with MLST, cgMLST and location with iTOL (*10*).

Limitations of Phylogeographic Analysis

While whole-genome sequencing provides high-resolution data, our conclusions regarding the outbreak's origin are subject to inherent limitations stemming from biases in the public genomic databases used for comparison. The global distribution of sequenced *B. pseudomallei* genomes is uneven due to geographic and sampling bias, meaning a more closely related strain may exist in a region with limited genomic surveillance. Furthermore, a temporal bias is evident in the significant time gap of ≈20 years and the 18-cgSNP genetic distance separating the Hong Kong Special Administrative Region, People's Republic of China isolates from their closest relatives, which indicates the direct ancestral lineage is unobserved. Finally, a source-type bias exists because public databases are heavily skewed toward human clinical isolates, with far fewer genomes from environmental or animal reservoirs. This suggests the outbreak could have originated from an unsequenced environmental source, a possibility consistent with our negative environmental sampling results at the zoo.

Reference

1. Wang H, Chen YL, Teng SH, Xu ZP, Xu YC, Hsueh PR. Evaluation of the Bruker Biotyper matrix-assisted laser desorption/ionization time-of-flight mass spectrometry system for identification of clinical and environmental isolates of *Burkholderia pseudomallei*. Front Microbiol. 2016;7:415.

2. Meumann EM, Novak RT, Gal D, Kaestli ME, Mayo M, Hanson JP, et al. Clinical evaluation of a type III secretion system real-time PCR assay for diagnosing melioidosis. J Clin Microbiol. 2006;44:3028–30.

3. Bouras G, Houtak G, Wick RR, Mallawaarachchi V, Roach MJ, Papudeshi B, et al. Hybracter: enabling scalable, automated, complete and accurate bacterial genome assemblies. Microb Genom. 2024;10:001244.

4. Godoy D, Randle G, Simpson AJ, Aanensen DM, Pitt TL, Kinoshita R, et al. Multilocus sequence typing and evolutionary relationships among the causative agents of melioidosis and glanders, *Burkholderia pseudomallei* and *Burkholderia mallei*. J Clin Microbiol. 2003;41:2068–79.

5. Jolley KA, Bray JE, Maiden MC. Open-access bacterial population genomics: BIGSdb software, the PubMLST.org website and their applications. Wellcome Open Research. 2018;3.

6. Shimoyama Y. ANIclustermap: A tool for drawing ANI clustermap between all-vs-all microbial genomes 2022 [cited 2025 Apr 2]. https://github.com/moshi4/ANIclustermap

7. Fang Y, Hu Z, Chen H, Gu J, Hu H, Qu L, et al. Multilocus sequencing-based evolutionary analysis of 52 strains of *Burkholderia pseudomallei* in Hainan, China. Epidemiol Infect. 2018;147:e22.

8. Seemann T. snippy: fast bacterial variant calling from NGS reads 2015 [cited 2025 Apr 2]. https://github.com/tseemann/snippy

9. Nguyen L-T, Schmidt HA, Von Haeseler A, Minh BQ. IQ-TREE: a fast and effective stochastic algorithm for estimating maximum-likelihood phylogenies. Mol Biol Evol. 2015;32:268–74.

10. Letunic I, Bork P. Interactive Tree of Life (iTOL) v6: recent updates to the phylogenetic tree display and annotation tool. Nucleic Acids Res. 2024;52:W78–82.

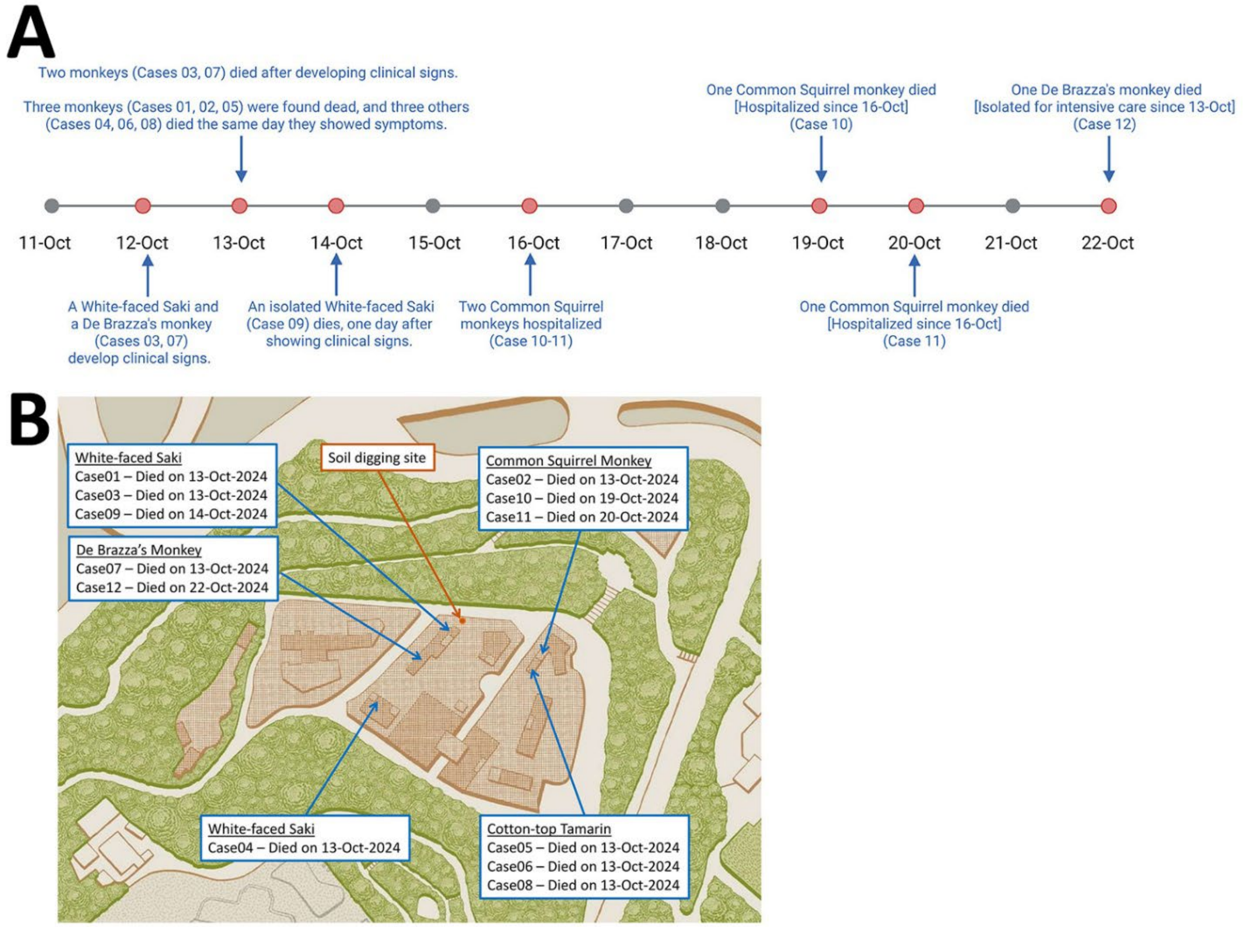

Appendix Figure 1. Temporal and spatial distribution of the primate melioidosis outbreak. (A) Timeline of clinical and epidemiologic events for the 12 cases. The timeline indicates the dates of symptom onset, hospitalization, and death for each case. (B) Map of the facility showing the spatial distribution of cases and the location of the environmental source. Arrows connect each case to their respective housing, and the soil digging site is marked with an orange arrow.

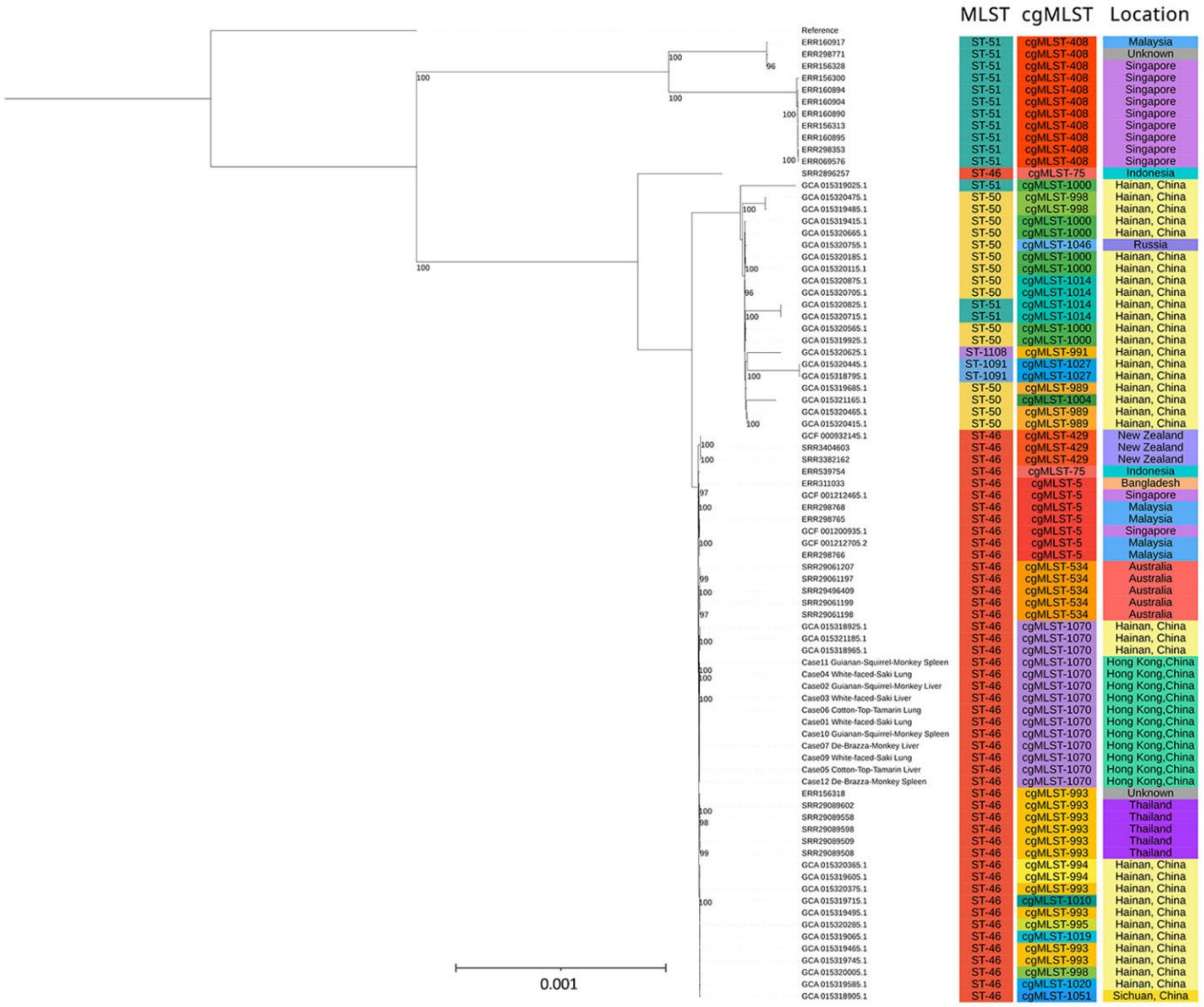

Appendix Figure 2. Phylogenetic analysis of 84 genomes of *B. pseudomallei* ST-46 (40 reference genomes acquired globally from NCBI and 11 genomes from monkey isolates collected from Hong Kong Special Administrative Region, People’s Republic of China in this study) and related sequence types, including ST-50, ST-51, ST-1091, and ST1108. Maximum-likelihood phylogenies of the SNPs of the 3,127 single-copy cgMLST gene is shown. The tree is mid-point rooted. Bootstrap analysis of 1,000 replicates was performed and bootstrap values of selected nodes are shown.

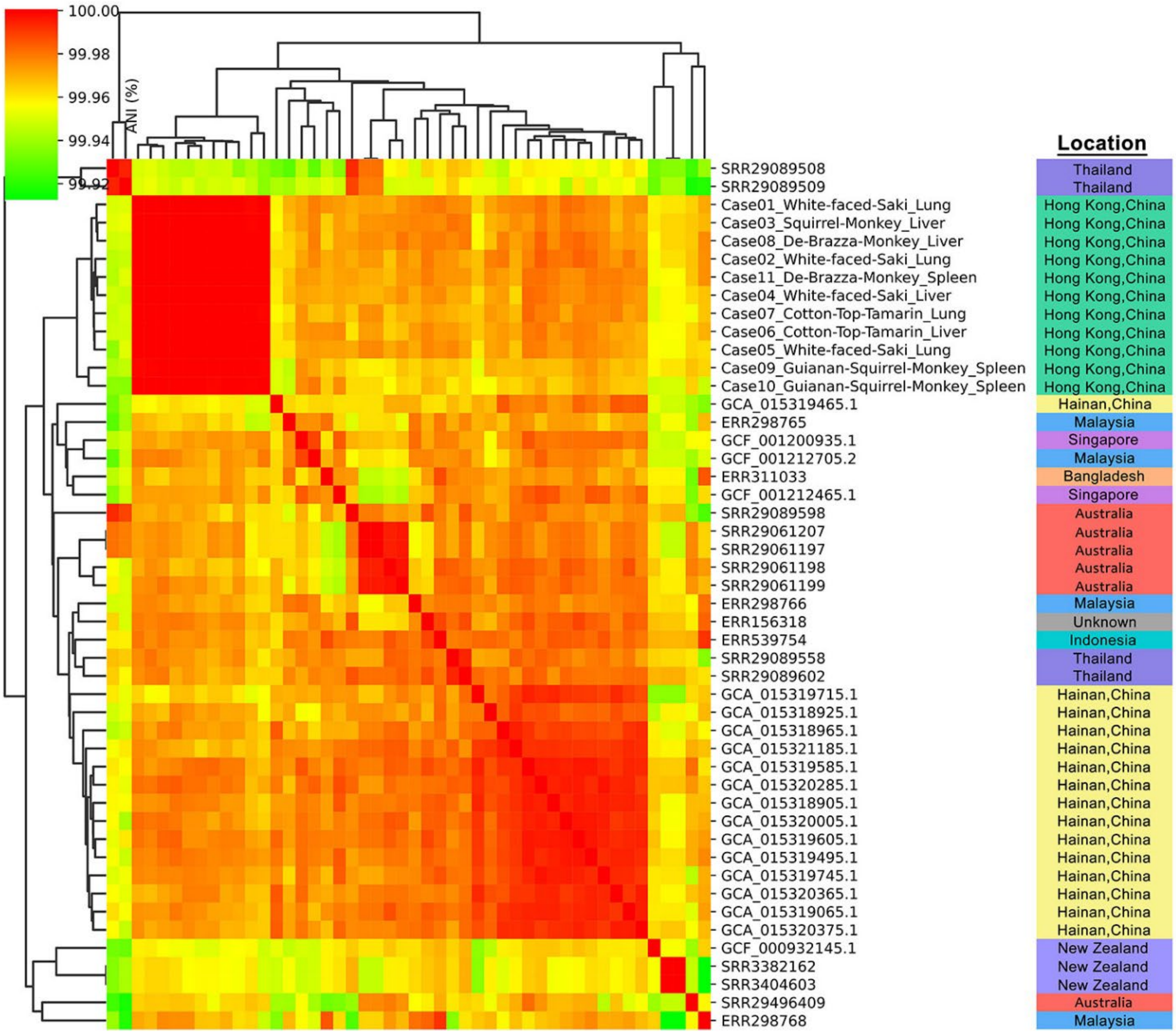

Appendix Figure 3. Hierarchical clustering of average nucleotide identity (ANI) among *B. pseudomallei* isolates. The heatmap displays whole-genome ANI values comparing the 11 primate isolates from the Hong Kong Special Administrative Region, People's Republic of China outbreak (Cases 01–11) with global ST-46 reference genomes. Color intensity represents ANI percentage (scale: 99.90%–100.00%), with red indicating highest similarity. UPGMA dendrograms show hierarchical clustering based on genetic relatedness.
